# Radiomics-based machine learning approach for the prediction of grade and stage in upper urinary tract urothelial carcinoma: a step towards virtual biopsy

**DOI:** 10.1097/JS9.0000000000001483

**Published:** 2024-05-03

**Authors:** Abdulsalam Alqahtani, Sourav Bhattacharjee, Abdulrahman Almopti, Chunhui Li, Ghulam Nabi

**Affiliations:** a School of Medicine, Centre for Medical Engineering and Technology; b School of Science and Engineering, University of Dundee, Dundee DD1 4HN, UK; c School of Veterinary Medicine, University College Dublin, Belfield, Dublin 4, Ireland; d Radiology Department, College of Applied Medical Sciences, Najran University, Najran 55461, Kingdom of Saudi Arabia

**Keywords:** CT urogram, machine learning, radiomics, texture analysis, virtual biopsy

## Abstract

**Objectives:**

Upper tract urothelial carcinoma (UTUC) is a rare, aggressive lesion, with early detection a key to its management. This study aimed to utilise computed tomographic urogram data to develop machine learning models for predicting tumour grading and staging in upper urothelial tract carcinoma patients and to compare these predictions with histopathological diagnosis used as reference standards.

**Methods:**

Protocol-based computed tomographic urogram data from 106 patients were obtained and visualised in 3D. Digital segmentation of the tumours was conducted by extracting textural radiomics features. They were further classified using 11 predictive models. The predicted grades and stages were compared to the histopathology of radical nephroureterectomy specimens.

**Results:**

Classifier models worked well in mining the radiomics data and delivered satisfactory predictive machine learning models. The multilayer panel showed 84% sensitivity and 93% specificity while predicting UTUC grades. The Logistic Regression model showed a sensitivity of 83% and a specificity of 76% while staging. Similarly, other classifier algorithms [e.g. Support Vector classifier (SVC)] provided a highly accurate prediction while grading UTUC compared to clinical features alone or ureteroscopic biopsy histopathology.

**Conclusion:**

Data mining tools could handle medical imaging datasets from small (<2 cm) tumours for UTUC. The radiomics-based machine learning algorithms provide a potential tool to model tumour grading and staging with implications for clinical practice and the upgradation of current paradigms in cancer diagnostics.

**Clinical Relevance:**

Machine learning based on radiomics features can predict upper tract urothelial cancer grading and staging with significant improvement over ureteroscopic histopathology. The study showcased the prowess of such emerging tools in the set objectives with implications towards virtual biopsy.

## Introduction

Highlights

- Computed tomographic imaging of upper tract urothelial carcinoma patients who underwent surgical radical nephroureterectomy were obtained. Images were segmented and data was extracted. The data were analysed using various algorithms.
- Data mining enabled *virtual biopsy* and predicted tumour grading and staging better than ureteroscopy biopsy. The reference standard was histopathology from radical surgery.
- The gained insights and knowledge have multiple implications for clinical practice and future research.

Upper tract urothelial carcinoma (UTUC) comprises 5–10% of all urothelial carcinomas^1^, has an incidence of 2/100 000 in the western population^2^, and is reported mostly in the age group of 70–90 years. While 95% of the urothelial carcinomas, growing from the inner lining of the urinary tract, relate to the urinary bladder^3^, they also originate in the renal pelvis and ureters, with the incidence of pelvicalyceal tumours almost twice that of ureteric ones^4^. Multifocal lesions are also noted in 10–20% of cases^5^, while 11–36% of cases are carcinoma *in situ* of the upper urinary tract^6^.

Ureteroscopic biopsy and urine cytology are known tools for confirming UTUC before surgical intervention^7^. In conjunction with biopsy, ureteroscopy provides crucial cues on tumour architecture, location, size, and focality^8–10^. Collectively, this information helps the surgeons decide on the management options. The diagnostic accuracy of ureteroscopic biopsy depends on the cancer grade, as reported in a recent systematic review^11^. The study reported an undergrading in 32% and an understaging in 46% of cases. Similarly, a recent multicentre study confirmed that tumour grading and staging are often inaccurate and vulnerable to understaging, including an inability to comment on grading in 31.5% of cases^12^.

Furthermore, visualising and accessing the upper urinary tract by ureteroscopy requires expertise and training^13^. The procedural challenges during ureteroscopic biopsy render many of the obtained samples unfit for histopathological evaluation due to contamination with crushed artefacts, making a pathological assessment difficult^14^. The sensitivity of urine cytology in detecting high-grade UTUCs can be high (84%), although it falls drastically to 16% in low-grade tumours^15^. Such limitations of biopsy and urine cytology in diagnosing the UTUCs risk overestimation or underestimation.

Computed tomography (CT) urogram can effectively diagnose UTUCs with sensitivity and specificity of 92 and 95%, respectively^16^. It is also more sensitive and specific in diagnosing UTUCs than the contrast magnetic resonance urogram, which has 75% sensitivity in detecting UTUCs <2 cm in size^17^. A CT-based visualisation of UTUCs demonstrates the microscopic overview of tumour anatomy in both 2D and 3D, with information on the extent of the tumour margin and its invasion towards adjoining tissues^18^.

Radiomics is an emerging field of medical imaging that involves extracting and analysing quantitative features from medical images, such as CT and magnetic resonance urogram, using machine learning algorithms that help to extract a large number of features and provide analyses^19^. Gathering such a large dataset of features allows modelling with implications for prognosis, progress review after radiotherapy or chemotherapy, and developing predictive models on clinical outcomes, including metastasis^20^. Furthermore, it identifies important diagnostic and therapeutic cancer biomarkers, including prediction of grade and stage^21^.

The present study evaluated the scope of radiomics analyses combined with machine learning in predicting the grade and stage of UTUCs compared to ureteroscopic biopsies. The corresponding histopathological data from radical nephroureterectomy were used as a reference standard.

## Patients and methods

### Patients

CT urogram and clinicopathological data of patients with UTUCs were obtained from the Tayside Urological Cancers (TUCAN) database. The data included UTUC patients who underwent surgical resection (radical nephroureterectomy) at a tertiary care hospital from January 2000 to December 2022 with a mean follow-up of 40 (±12–120) months. The study received approval from the East of Scotland Research Ethical Service (Approval No. IGTCAL12952). Access to patient medical healthcare data was granted under the Caldicott Approval, and the requirement for informed consent was waived. The study inclusion criteria were:

- Availability of protocol-based CT urogram images.
- Pathologically confirmed UTUC with uniform criteria for determination, specifically obtained after surgical resection and all patients underwent ureteroscopic biopsy prior to surgery.
- No prior endoscopic treatment of UTUC before CT examination.

**Figure 1:**
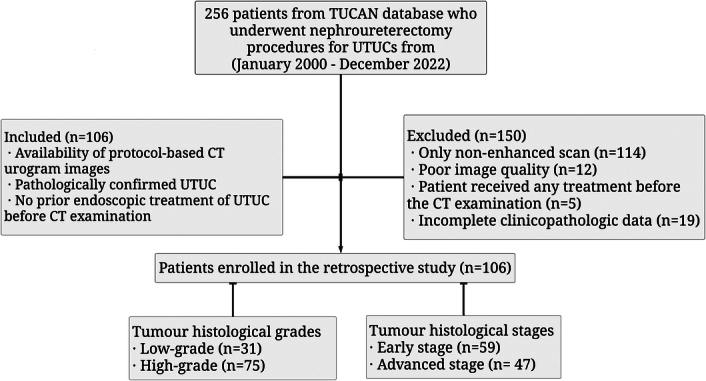
Workflow showing the flow of patient selection strategy followed in this study.

Of the 256 patients subjected to nephroureterectomy for UTUCs in the TUCAN database, 106 (consecutive cases) met the inclusion criteria. Concurrently, 150 patients were excluded from the study due to various criteria, including nonenhanced scans (114), inadequate image quality (12), prior CT treatment (5), and 19 for lacking comprehensive clinicopathologic data (Fig. 1). The tumour histological grades in the dataset were categorised into low-grade (I–II) in 31 cases and high-grade (III–IV) in 75 cases. Similarly, the tumour histological stages were classified as early-stage (Ta–T1) in 59 cases and advanced-stage (T2–T4) in 47 cases. The pathology data was verified by a uropathologist with more than 10 years of experience, and each case was discussed in a tumour board meeting. For further details and CT protocol, please see Supplementary Material S1 (Supplemental Digital Content 1, http://links.lww.com/JS9/C474). The study was conducted in line with the Standards for the Reporting of Diagnostic Accuracy Studies (STARD) criteria^22,23^ (Supplemental Digital Content 2, http://links.lww.com/JS9/C475).

### Tumour segmentation using 3D Slicer

The methodology employed in this study included a series of procedures to effectively handle Digital Imaging and Communications in Medicine (DICOM) images, perform 3D segmentation^24^ utilising the ‘grow-from-seeds’ tool of the 3D Slicer (version 5.2.2), and convert the DICOM slices into the widely adopted 3D Neuroimaging Informatics Technology Initiative (NIfTI) format. The segmentation task was performed by an experienced radiologist (Reader 1) and an expert urosurgical oncologist (Reader 2). To ensure the precision of segmentation, a Dice similarity coefficient was applied, maintaining a 0.8 threshold as the benchmark to ensure a minimum of 80% overlap accuracy. Segmentations satisfying this criterion progressed to subsequent analyses. In scenarios where segmentations fell short of the threshold, a collaborative discussion was initiated between Readers 1 and 2 to identify areas that required refinement to arrive at a consensus. If such a mutual agreement remained elusive, the case was excluded. The histopathological evaluation following nephroureterectomy was considered the reference standard (Supplementary Materials S1, Supplemental Digital Content 1, http://links.lww.com/JS9/C474 and S2, Supplemental Digital Content 1, http://links.lww.com/JS9/C474).

### Feature extraction

**Figure 2:**
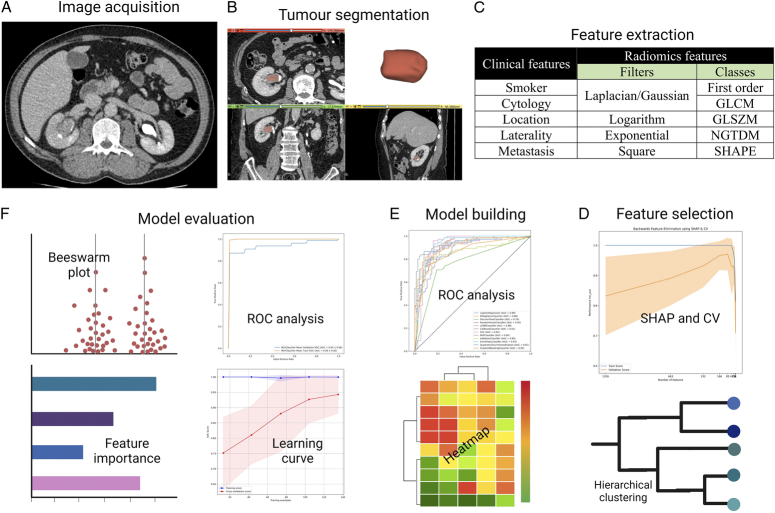
The workflow of the radiomics analysis and machine learning. (A) High-quality CT urogram images were acquired and preprocessed; (B) A ROI was segmented, encompassing the entire tumour, and rendered in 3D; (C) Feature extraction; (D) Feature selection; (E) Combined model development based on radiomics features and multiple clinical variables; (F) Model evaluation. ROI, region of interest.

Quantitative features were extracted from segmented regions of interest using the PyRadiomics library in Python software (v. 3.7.9). An in-house algorithm was scripted for radiomics analyses and model building. It allowed the extraction of quantitative features, feature selection, and integration of radiomics data with clinical variables to create a predictive model. Additionally, it facilitated performance evaluation and feature contribution analysis of the optimal prediction model. A significance level of *P*<0.05 was considered statistically significant (Fig. 2).

Three feature classes were computed: first-order statistics, texture-based, and shape-based features. First-order statistics reflected intensity distribution; texture-based features represented textural properties using matrices like Grey-Level Co-occurrence Matrix, Grey-Level Run-Length Matrix, and Grey-Level Size-Zone Matrix, while shape-based features assessed geometric properties. All feature classes were calculated using various filters in the PyRadiomics package, resulting in 1324 features. To address the class imbalance issue, the Synthetic Minority Over-sampling Technique (SMOTE) was utilised to oversample the low-grade class. The study workflow is summarised in Figure 2.

### Feature stability and selection

This study commenced with a meticulous data preparation phase, where certain variables, particularly clinical and non-numeric ones, were identified and exempted from normalisation. The remaining numeric variables underwent Z-score normalisation for setting the standards. A crucial step involved performing a correlation analysis to eliminate highly correlated variables (>0.9 threshold) and ensure statistical independence. The dataset was then categorised and segmented into two distinct groups: clinical and radiomics variables, facilitating targeted analyses for each group. A comprehensive dataset overview was presented, including detailed variable listings for both clinical and radiomics datasets.

In the initial phase, attributes derived from segmentations performed by Readers 1 and 2 were subjected to an inter-rater reproducibility assessment. Attributes demonstrating an Intraclass Correlation Coefficient (ICC) of <0.75 were filtered, resulting in 163 features for further analysis. The backward feature elimination using shape and cross-validation (CV) was employed to select the optimal feature set. The process involved estimating SHapley Additive exPlanations (SHAP) feature importance, removing low-importance features, and visualising the results through plots. This approach facilitated effective feature selection based on the impact on model performance (Fig. 2).

### Evaluation of the constructed prediction model and statistical analysis

A power analysis, using a Goodness-of-fit Chi-square power analysis suitable for the binary nature of our target variables (high-grade vs. low-grade), was conducted to validate the sample size. An effect size of 0.5 (Cohen’s w), a significance level of 0.05, and a power level of 80% were assumed. Multiple machine-learning models were evaluated. In each model, five-fold CV (k=5) with hyperparameter optimisation was performed. Performance metrices such as area-under-curve (AUC), sensitivity, specificity, 95% CI, classification accuracy, F1 score, precision, and recall were used to assess model performance. Receiver Operating Characteristic (ROC) curves were plotted for model comparison. One-way ANOVA determined the best-performing dataset. Box plots and bar charts were used to compare the performance of different UTUC grade and stage models. Further analysis included ROC AUC, confusion matrices, learning curves, feature importance ranking using SHAP values, and Beeswarm plots to examine the performance of the best model within the best dataset.

## Results

### Patient characteristics

The study included 106 patients, and Table 1 shows a detailed characterisation of the patient cohort.

**Table 1:** Overview of patient characteristics and tumour features in the entire cohort (*n*=106).

Mean age	Sex (%)		Smoker (%)		BMI (%)		Cytology (%)		Deceased (%)		Metastasis (%)		CTU findings (%)		Laterality (%)		Location (%)		Histology grade (%)		T-stage (%)	
74 yrs. (49–93 yrs.)	M	58.5	Never	23.6	Normal	33	Positive	20.8	Y	54.7	Y	11.3	Y	100	Left	40.6	Renal pelvis	56.6	Low	29.2	Ta/T1	55.7
	F	41.5	Smoker	30.2	Overweight	34	Negative	29.2	N	45.3	N	88.7	N	0	Right	59.4	Ureter	62.3	High	70.8	T2	17.9
			Ex-smoker	46.2	Obese	33	Suspected	9.4							T3/T4	26.4						
							Undiagnosed	40.6		CIS	23.6											

CIS, carcinoma *in situ;* CTU, CT urogram.

### Radiomics feature selection

The selection of radiomic features for tumour grading and staging leveraged diverse families derived from CT imaging to detail the tumour’s complexity. This encompassed features from image transformations such as wavelet, square, and gradient—alongside metrices of shape and texture.

### Radiomics models for tumour grading

**Figure 3:**
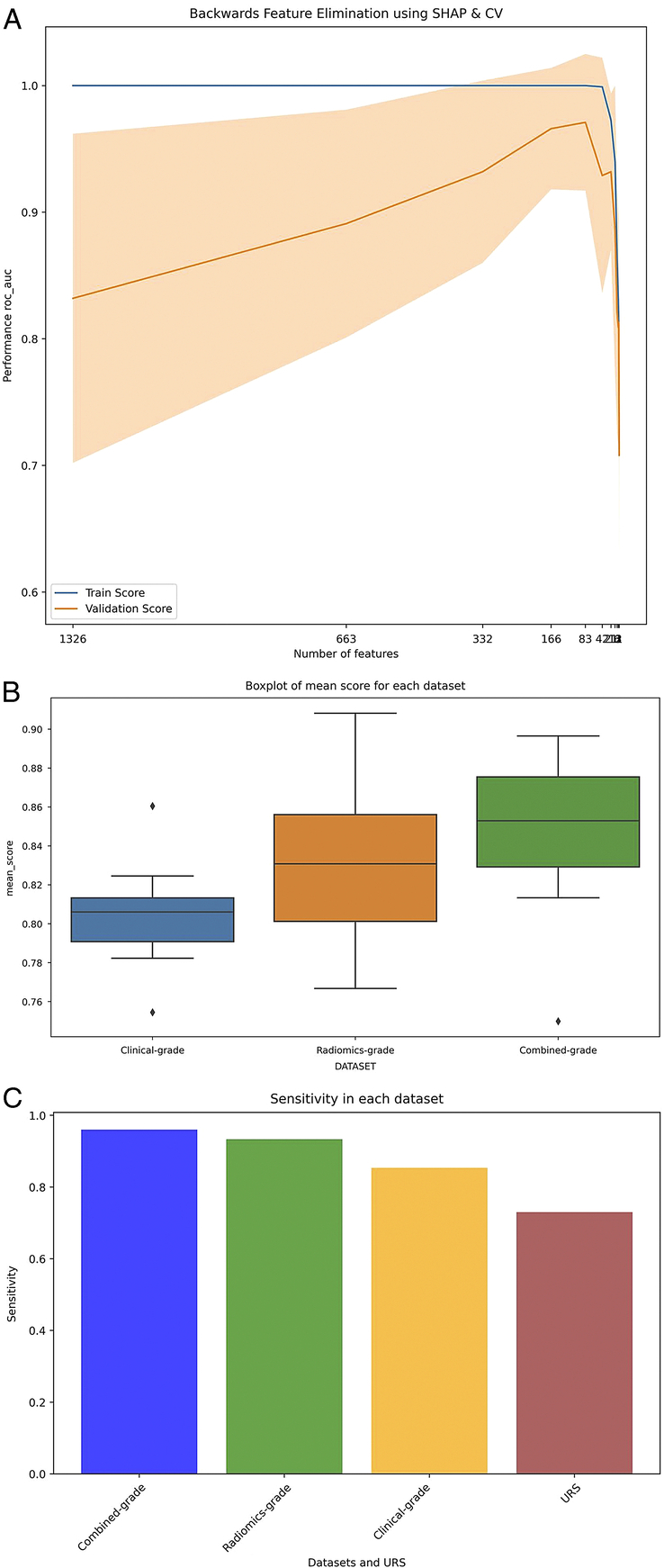
Radiomics models for tumour grading. (A) The utilisation of RFECV with SHAP feature importance and cross-validation; (B) Box plot demonstrating that the combined-grade dataset outperformed other datasets with the highest mean score; (C) Bar chart enabling comparison of UTUC grade dataset performance across multiple classification models providing valuable insights while assessing the ureteroscopic biopsy dataset with UTUC grades. RFECV, recursive feature elimination with cross-validation; SHAP, Shapley additive explanations.

The range for tumour volume and size (*n*=106) varied between 0.03–167.52 cm^3^ and 7 mm–13 cm, respectively. The power analysis confirmed the adequacy of our sample size. With 106 patients, the study exceeded the minimum requirement of ~32 patients per group to achieve the desired statistical power of 80%. Eleven classification models were used to differentiate high-grade from low-grade tumours and early TNM stages from the advanced ones. Features were selected using Recursive Feature Elimination, identifying 11 informative features for radiomics and combined datasets (Fig. 3A). Mean scores of metrices across datasets were compared. The combined-grade dataset had the highest mean score (M=0.85), followed by the radiomics-grade (M=0.83) and clinical-grade datasets (M=0.8). No significant difference was found between radiomics-grade and combined-grade datasets (*P*=1.0) or between radiomics-grade and clinical-grade datasets (*P*=0.27). However, a significant difference was noted between combined-grade and clinical-grade datasets (*P*=0.02; Fig. 3B). In assessing the effectiveness of 11 predictive models for UTUC tumour grading, the study delineated them into three groups based on statistical performance metrics. Notably, the SVC and multilayer panel (MLP) exhibited superior performance, showcasing exceptional precision, recall, F-score, and ROC AUC values. SVC demonstrated remarkable sensitivity at 96.3%, highlighting its potential for accurate high-grade tumour identification. MLP, on the other hand, demonstrated an ROC AUC value of 0.94, indicating its overall predictive accuracy and reliability. Meanwhile, CatBoost, ExtraTrees, Quadratic Discriminant Analysis (QDA), and Gradient Boosting offered moderate efficacy, showcasing a reliable balance in sensitivity and specificity. Conversely, models including Logistic Regression, KNeighbors, Decision Tree, Random Forest, Light Gradient Boosting Model (LGBM), and AdaBoost revealed areas requiring improvement, underscoring the potential for enhanced model performance through further advancements in UTUC grading methodologies (Table 2). Ureteroscopic biopsies predicted histological grade with an accuracy of 77.27%, sensitivity of 72.73%, and specificity of 86.36%. The match rates for low-grade and high-grade cases were 61.29 and 91.43%, respectively, Supplementary Material S2 (Supplemental Digital Content 1, http://links.lww.com/JS9/C474). Using a combined dataset effectively discriminated between high-grade and low-grade tumours (Fig. 3C). For hierarchical clustering of the cohort, please consult Supplementary Materials S3 (Supplemental Digital Content 1, http://links.lww.com/JS9/C474).

**Table 2:** Performance of classification models of the combined grade dataset.

Classifier model	Precision	Recall	F-score	ROC AUC	95% CI	Sensitivity	Specificity
Logistic Regression	0.83	0.83	0.83	0.9	0.84–0.95	80%	86.6%
KNeighbors	0.84	0.83	0.83	0.87	0.8–0.92	73.3%	92.5%
Decision Tree	0.75	0.75	0.75	0.77	0.69–0.84	70.6%	78.6%
Random Forest	0.83	0.83	0.83	0.92	0.87–0.97	80%	86.6%
LGBM	0.8	0.81	0.8	0.88	0.82–0.94	78%	81.3%
CatBoost	0.85	0.85	0.85	0.91	0.86–0.96	80%	89.3%
SVC	0.9	0.89	0.89	0.92	0.87–0.96	96.3%	81.3%
MLP	0.9	0.89	0.89	0.94	0.9–0.98	84%	93.3%
AdaBoost	0.81	0.81	0.81	0.85	0.79–0.91	77.3%	84%
ExtraTrees	0.85	0.85	0.85	0.93	0.89–0.97	78.6%	90.6%
QDA	0.89	0.89	0.89	0.91	0.87–0.96	86.6%	90.6%
Gradient Boosting	0.86	0.86	0.86	0.92	0.87–0.97	82.6%	89.3%

LGBM, light gradient boosting model; MLP, multilayer panel; QDA, quadratic discriminant analysis; SVC, support vector classifier.

### Performance evaluation of features and building prediction model

**Figure 4:**
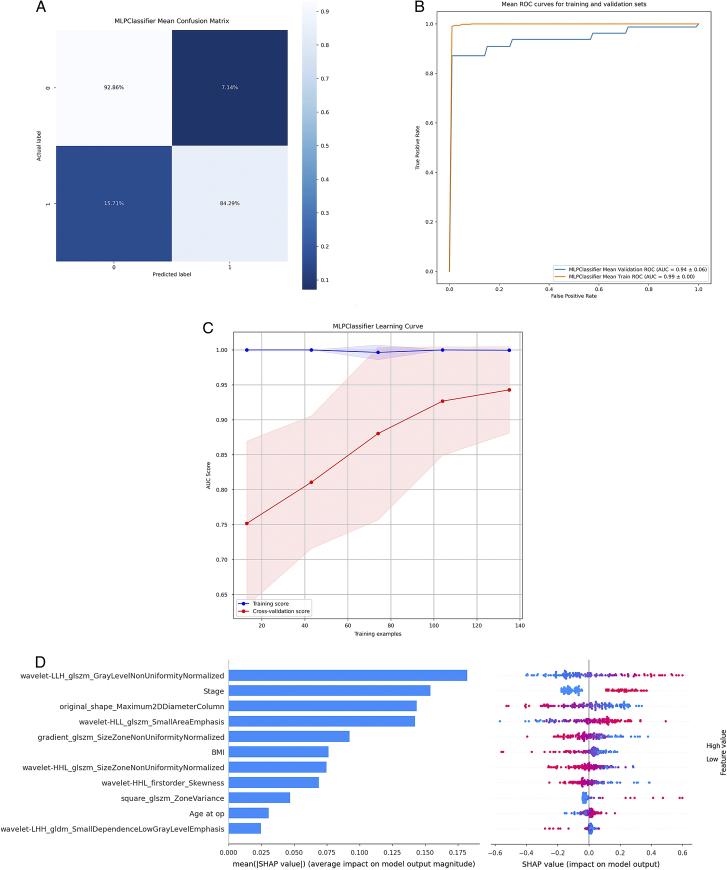
Performance of the proposed model in the testing set. (A) ROC curve of the proposed model in pathological grade prediction; (B) Confusion matrix showing the performance of the MLP model in classifying two classes: H and L; (C) The learning curve for predicting high and low-grade UTUCs with AUC scores on the *y*-axis and number of training samples on the *x*-axis; (D) Feature importance ranking based on SHAP values generated from the test set; (E) A Beeswarm plot displaying the Shapley values of features in the final model. Each feature value is represented by coloured dots: red for high-grade and blue for low-grade tumours. H, high; L, low; ROC, receiver operating characteristic.

The MLP Classifier model showed high accuracy with 92.86% sensitivity and 84.29% specificity on the testing set (Fig. 4A). It demonstrated excellent discrimination and generalisation abilities with an AUC of 0.99 on the training and 0.94 on the testing set (Fig. 4B). The learning curve indicated convergence and potential performance improvement with more training data (Fig. 4C). SHAP analysis provided insights into feature significance, with stage and BMI having notable impacts (Fig. 4D, Supplementary Materials S2, Supplemental Digital Content 1, http://links.lww.com/JS9/C474).

### Comparative analysis of classifier models for UTUC stage prediction

**Figure 5:**
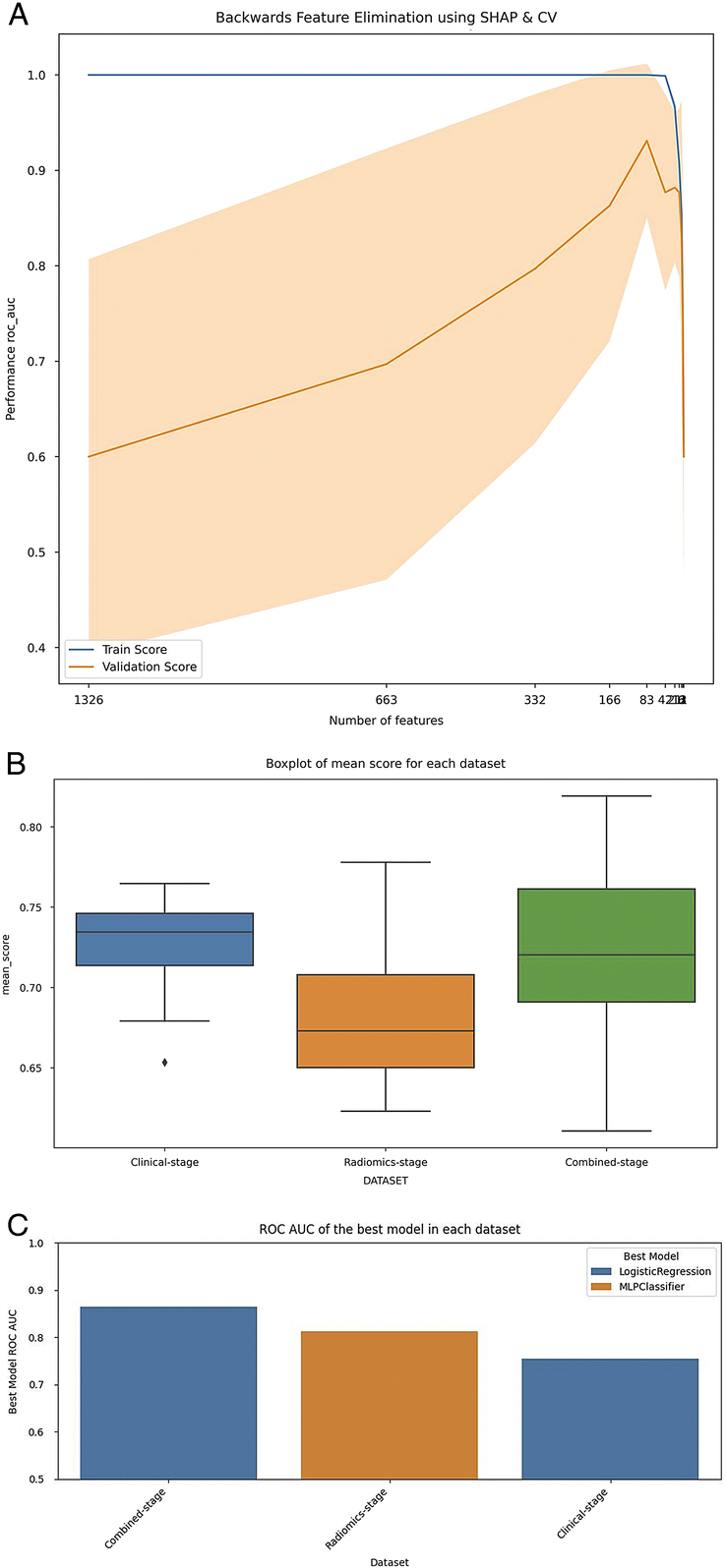
Radiomics models for tumour staging. (A) RFECV with SHAP feature importance and cross-validation was used to identify optimal features and feature sets; (B) Box plot demonstrating that the combined-stage dataset performed best, with the highest mean score across the evaluated metrices; (C) Bar chart enabling comparison of UTUC stage dataset performance across multiple models. RFECV, recursive feature elimination with cross-validation; SHAP, Shapley additive explanations.

Eleven classification models were applied to clinical variables, radiomics, and combined datasets. Using recursive feature elimination with SHAP and CV (Fig. 5A), the combined stage dataset showed a superior ability to differentiate between early and advanced stages compared to clinical-based and radiomics-based staging. The clinical stage had the highest mean score (M=0.72), followed by the combined (M=0.72) and the radiomics stages (M=0.68). No significant difference was noted between the combined and radiomics stages (*P*=0.34) or combined and clinical stages (*P*=1.0), although significance between radiomics and clinical stages (*P*=0.1; Fig. 5B) was identified.

This study aimed to evaluate eleven predictive models for UTUC staging and categorised them into three groups. The study revealed that the Logistic Regression model emerged as the top performer with an F-score of 0.8 and an impressive ROC AUC of 0.86, demonstrating an optimal balance between precision and recall. The model achieved sensitivities and specificities of 83 and 76.2%, respectively, highlighting its robustness for clinical use in UTUC staging (Fig. 5C). The second group included MLP, SVC, and ExtraTrees classifiers, with MLP exhibiting a notable ROC AUC of 0.84 and SVC being highlighted for its precision in identifying tumour stages with 77.6% sensitivity and 77.9% specificity. The ExtraTrees classifier also demonstrated adequate predictive prowess, with 83% sensitivity. However, the third group, which featured models such as Decision Tree, Random Forest, Light Gradient Boosting Model (LGBM), and AdaBoost, indicated a need for improvement in sensitivity, specificity, or ROC AUC scores, suggesting areas for potential refinement (Table 3).

**Table 3:** Performance of classification models of the combined stage dataset.

Classifier model	Precision	Recall	F-score	ROC AUC	95% CI	Sensitivity	Specificity
Logistic Regression	0.8	0.8	0.8	0.86	0.8–0.93	83%	76.2%
KNeighbors	0.75	0.74	0.73	0.73	0.64–0.82	83%	64.4%
Decision Tree	0.62	0.62	0.62	0.57	0.47–0.67	62.7%	61%
Random Forest	0.69	0.69	0.69	0.74	0.66–0.83	71.1%	66.1%
LGBM	0.69	0.69	0.69	0.73	0.65–0.83	71.1%	66.1%
CatBoost	0.7	0.69	0.69	0.74	0.65–0.83	77.9%	61%
SVC	0.78	0.78	0.78	0.79	0.71–0.87	77.6%	77.9%
MLP	0.82	0.81	0.81	0.84	0.77–0.92	86.4%	76.2%
AdaBoost	0.64	0.64	0.63	0.67	0.56–0.76	59.3%	67.7%
ExtraTrees	0.76	0.75	0.75	0.75	0.66–0.84	83%	67.7%
QDA	0.73	0.73	0.73	0.78	0.69–0.86	79.6%	66.1%
Gradient boosting	0.68	0.68	0.68	0.69	0.6–0.79	71.1%	64.4%

LGBM, light gradient boosting model; MLP, multilayer panel; QDA, quadratic discriminant analysis; SVC, support vector classifier.

### Performance evaluation and features contribution analysis

**Figure 6:**
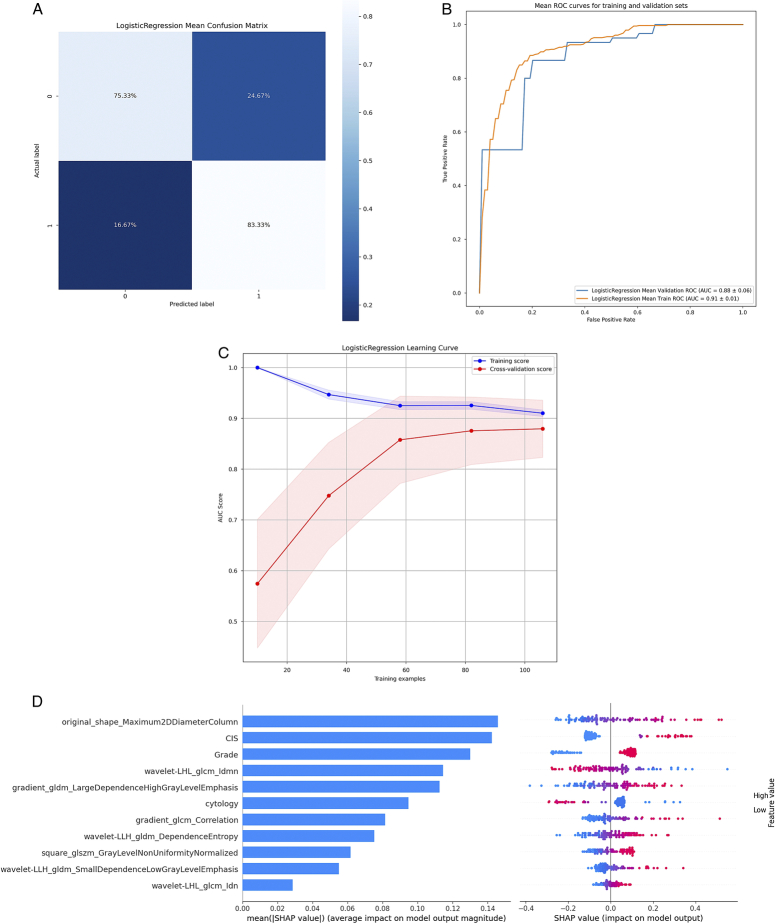
Performance of the proposed model in the testing set. (A) Confusion matrix showing the performance of the logistic regression model in classifying two classes: H and L; (B) ROC curve of the proposed model in pathological stage prediction; (C) The learning curve for predicting high and low-stage UTUCs with AUC scores on the *y*-axis and number of training samples on the *x*-axis; (D) Feature importance ranking based on SHAP values generated from the test set; (E) A Beeswarm plot displaying the Shapley values of features in the final model. Coloured dots represent each feature value: red for an advanced-stage and blue for early-stage tumours. H, high; L, low; ROC, receiver operating characteristic.

The Logistic Regression classifier showed high accuracy, with a sensitivity of 75.33% and specificity of 83.33% (Fig. 6A). It demonstrated excellent discrimination and generalisation, achieving an AUC of 0.91 on the training and 0.88 on the testing set (Fig. 6B). Additional training data could improve its performance (Fig. 6C). SHAP analysis highlighted their significance and the impact of other features on data modelling (Fig. 6D, Supplementary Materials S3, Supplemental Digital Content 1, http://links.lww.com/JS9/C474).

## Discussion

Like many other cancers, early detection is key to a better prognosis and devising more effective treatment options in UTUCs^25^. However, histopathological evaluation, considered the gold standard in cancer detection, suffers from a paucity of data, empirical variation, and a lack of 3D visualisation. Advanced imaging techniques, such as CT and MRI, provide a wealth of information otherwise impossible by traditional 2D histopathology. It is prudent to note here that the complex datasets delivered by such advanced imaging modalities also need thorough analysis, and that is where a radiomics-based machine learning approach holds its major advantages^26^.

The effectiveness of a machine learning model in predicting the tumour grade and stage of UTUC was demonstrated by a notable improvement in sensitivity of 84% and specificity of 93% compared to traditional biopsy methods. This enhancement can be attributed to several factors. Clinically, UTUC presents unique challenges for biopsy due to its anatomical and pathological characteristics, such as its multifocality and potential for small, scattered lesions, leading to sampling errors. Our approach integrates advanced radiological techniques with a sophisticated machine learning algorithm, enabling a more comprehensive analysis of the CT data. This combination allows for the detection of subtle tissue differences and provides a global assessment of the area, surpassing the localised insight offered by biopsy samples. The model leverages a variety of data inputs, including texture features and patient demographics, processed through algorithms, and had undergone rigorous training and testing to ensure reliable predictions. Our findings are further supported by a review of existing literature, highlighting the limitations of biopsies in accurately staging and grading UTUC and similar cancers.

CT scan data provide both 2D and 3D textural analysis of the tumour parenchyma and the geometric attributes or other features now routinely extracted as part of data mining on medical datasets^27,28^. It is necessary to appreciate the prowess of data mining that can find patterns ingrained within the imagery data and then inform predictive models based on it^29^. Progress on the fronts of artificial intelligence-based platforms has brought fresh hopes that such emerging modalities will be able to replace human errors in cancer diagnostics in alliance with improved accuracy. Moreover, an advanced textural assessment can reveal the nascent foci of metaplasia or dysplasia within tissue fabric that are difficult to detect by routine histopathology^30,31^.

Comparable previous studies on UTUC radiomics and textural analyses are scarcely reported. Although not entirely comparable to ours, a recent study reported the use of texture analysis in evaluating tumour tissue heterogeneity in 86 UTUC cases based on CT datasets^32^. Interestingly, the histogram from CT attenuation from the tumours showed a single peak for noninvasive tumours, whereas multiple peaks were detected in tumours that were invasive towards the neighbouring muscular tissue. Such discrepancy may be attributed to the tumour microenvironment in UTUCs that evolves with its invasive potential.

The rarity of UTUCs is a challenge for such a study, as it is difficult to gather an adequately large cohort of patients for data analysis^28^. For the same reasons, the dataset used here, despite being collected over 22 years, is also insufficient and renders external validation difficult—an undeniable weakness of the present study. Moreover, the UTUC tissue often casts similar grayscale values to the adjoining healthy renal tissue in CT investigations, making a digital segmentation of the tumour out of the renal mass or extracting radiomics features cumbersome. Using contrast CT may be a strategy to address the issue, as was done in the present study.

It utilised advanced machine learning techniques to select radiomic features, bypassing subjective bias and emphasising features most relevant to the underlying tumour biology. Most selected features, predominantly second-order statistics and those pertaining to shape and other distinct families, offered a profound representation of the tumour’s biological complexity. These features are instrumental in capturing the textural and geometric heterogeneity of tumours and are critical in assessing tumour grade and stage. This selection process aligned with radiomics research principles that recommend limiting feature inclusion to one feature per 10 subjects. Such constraint ensures that only the most discriminative features are included, enhancing the model’s ability to maximise the ROC curve while minimising the risk of overfitting. Various classification models were used in this study, and overall, their performances were comparable, with some having an edge over others. It reiterates the primordial caution while developing any prediction model that the prediction quality is only as good as the training data. The present study included patients with a prior agreed CT urogram protocol.

In the present study, the clustering tools (Supplementary Material S3, Supplemental Digital Content 1, http://links.lww.com/JS9/C474) and algorithms (MLP for grading and Logistic Regression for staging) could segregate the high-grade UTUCs from the low-grade and early-stage tumours from the advanced ones. It is an exciting observation because of the following reasons:

- The methodology showed the individual clusters along with their memberships that, in a way, created a data matrix where the similarity, or its lack thereof, between different patients became apparent;
- It provided a necessary template where outliers could be traced, for example, a seemingly odd appearance of high-grade tumour in a cluster mostly populated by low-grade ones and *vice versa*, raising warning flags for the medical team to trace back and cross-check the sample;
- An offshoot of the clustering modules was the grading prediction in unknown UTUCs, where it would be interesting to note how the algorithm clustered the unknown instances, that is, high-grade or low-grade and early-stage or advanced-stage tumours.

To our knowledge, this study is the first systematic study investigating the scope of implementing a radiomics-based approach and machine learning tools in UTUCs for predicting the grade and stage. In a multicentre study, prenephroureterectomy biopsy histopathology correctly matched grade only in 43.4% of the histology findings. A significant number of cases (39.1%) were upstaged^12^. Such mismatch was much lower in the radiomics-based machine-learning approach reported here. With the current challenges faced by the healthcare sector, including a lack of resources and trained personnel, embracing the fruits of such emerging diagnostic modalities is a call of time to provide cancer diagnostics that are reliable, affordable, robust, brisk, high-throughput, and reproducible. Moreover, such tools and the digitisation of datasets enable the sharing of wisdom over continental stretches, with its influence spread across the planet and benefits shared by the entire humanity. The study findings—noninvasive prediction of UTUC aggressivity through radiomics and machine learning approach—have significant implications for clinical practice and lead to a new area of knowledge that has enormous benefits due to quicker and better decision-making for patients with upper tract urothelial cancers. As these technologies integrate into clinical practice, they may eventually replace traditional diagnostics, advancing the field towards personalised medicine in UTUC care.

To counter the challenge posed by a limited dataset of 106 cases, methodological strategies were deployed to enhance model reliability and mitigate overfitting. Employing k-fold CV optimised data during training and validation, while regularisation (L1 and L2) and early stopping minimised model complexity and prevented overtraining. Feature selection was confined to a maximum of 12, based on stability through the ICC, alongside normalisation and imbalance adjustments. The application of binary classification and hyperparameter tuning across diverse models ensured the robustness of the findings, avoiding model-specific bias. These precautions substantially mitigate overfitting, with future work to include external dataset for validating model effectiveness.

This study’s retrospective nature introduces potential biases, such as selection and information bias, which might impede the generalisation of our findings. These biases emerge from relying on pre-existing records and may not fully represent the broader UTUC patient population. Additionally, the quality and completeness of historical data can vary, potentially affecting the study’s accuracy. Our findings underline the importance of prospective multicentre trials for validation across diverse and larger datasets to curb these biases. A significant limitation of this study is the modest sample size, constrained by the rarity of UTUC. To strengthen our radiomics-based model, future research should focus on larger, prospective studies with standardised data that reflect a wider clinical spectrum.

## Conclusion

This study introduces a radiomics-based machine learning approach for UTUC, offering a method to accurately predict tumour grade and stage noninvasively. It demonstrates that classifier models can reliably differentiate high and low grades, as well as advance from the early stages of UTUC—thus providing a level of precision akin to traditional biopsies. The implementation of enhanced CT urogram and machine learning advancements marks the advent of ‘virtual biopsies’, providing a safer and more efficient tumour characterisation method. This noninvasive technique holds the potential to revolutionise oncology patient management and treatment planning, particularly for UTUC. By enabling precise tumour grading and staging presurgery, clinicians can customise treatment plans more effectively, potentially enhancing patient outcomes. The integration of these diagnostic tools could also improve the detection of small or indistinct tumours, facilitating earlier intervention.

## Ethical approval

The diagnostic study used images of patients stored in a hospital archives. The permission to use was granted by Caldicott approval (no IGTCAL12952 dated 25/10/2021).

## Consent

The Caldicott approval covers consent and hence no separate consent was necessary.

## Sources of funding

Abdulsalam Alqahtani was funded by Kingdom of Saudia Arabia for his doctoral studies.

## Author contribution

G.N. and A.A.: study concept or design; A.A. and A.A.: data collection; A.A., C.L., and S.B.: data analysis or interpretation; A.A., G.N., C.L., A.A., and S.B.: writing the paper.

## Conflicts of interest disclosure

All authors declare no conflicts of interest.

## Research registration unique identifying number (UIN)

1. Name of the registry: not applicable.
2. Unique identifying number or registration ID: not applicable.
3. Hyperlink to your specific registration (must be publicly accessible and will be checked): not applicable.

## Guarantor

Professor Ghulam Nabi. E-mail: GNabi@dundee.ac.uk

## Data availability statement

All the data are available on request for sharing with groups or with central repository following the University of Dundee policy and procedures.

## Provenance and peer review

Not applicable.

## Supplementary Material
