# Supplementary material for: Radiomics-based machine learning approach for the prediction of grade and stage in upper urinary tract urothelial carcinoma: a step towards virtual biopsy
Source: Int J Surg. 2024 May 3;110(6):3258–68. doi: 10.1097/JS9.0000000000001483 (PMC11175789; doi:10.1097/JS9.0000000000001483)
Supplement: Supplementary file 1 [file js9-110-3258-s001.docx]

**Supplementary Material S1. Patients and methods—Detailed overview**

*Patients*

A retrospective evaluation of prospectively maintained TUCAN data was carried out. The data included patients who underwent surgical resection for UTUC at a tertiary care hospital from 2000–2022 CE. The study received approval from the East of Scotland Research Ethical Service. Access to patients’ medical healthcare data was granted under the Caldicott Approval Number, and the requirement for informed consent was waived. The inclusion criteria were protocol-based specific CT urogram image availability and pathologically confirmed UTUC. No patient received any treatment before the CT examination. Out of the 256 patients in the institutional registry, 106 met the inclusion criteria. The imaging data utilised in the study was obtained from the Picture Archiving and Communication System (PACS). The data was provided in de-identified Digital Imaging and Communications in Medicine (DICOM) format, with a 512 × 512 × 3-pixel resolution. The demographic characteristics of the patients were documented and recorded. The tumour histological grades in the dataset were categorised into low-grade (I–II) in 31 cases and high-grade (III–IV) in 75 cases. Similarly, the tumour histological stages were classified as early-stage (Ta–T1) in 59 cases and advanced-stage (T2–T4) in 47 cases. The data used in this study were obtained from medical records following surgical resection (Figure 1).

*CT protocol*

Images from the group were obtained using a GE Healthcare Helical CT scanner. The scan parameters included a large body Scan Field of View (SFOV), 0.7 s gantry rotation time, 1.25 mm slice thickness, 1375:1 pitch, 40 mm detector coverage, Noise Index (NI) of 30, and CT Dose Index Volume (CTDIvol) 9.59 mGy. The X-ray tube voltage was set to 120 kVp, and the X-ray tube current was automatically adjusted based on the patient’s size. The scan protocol includes a non-contrast-prone KUB. 90 mL of contrast was injected over 15 min for contrast-enhanced images. Supine Abdo/Pelvis pictures were taken in 100 s, with 60 mL of contrast injected at a rate of 3mL/s.

*3D Slicer*

The 3D Slicer software, freely downloadable from the website http://www.slicer.org, is an interactive, open-source, and freely available software for medical image analysis and 3D visualisation. It can handle multiple medical image file formats, including the DICOM format, the most widely used and considered the industry standard in medical image format. Besides batch processing of imagery datasets, it can handle 2D, 3D, and 4D datasets and is compatible with Windows, macOS, and Linux operating systems. One of its key features is segmentation, where a volume of interest, such as a space-occupying lesion like a tumour, can be isolated from a 3D-rendered scene for further analysis. Furthermore, 3D Slicer offers options for manual editing a 3D rendered scene and has been used for multiple clinically relevant instances, including lung, prostate, and breast cancers, orthopaedic and neurosurgery, multiple sclerosis, cardiovascular ailments, and chronic obstructive pulmonary diseases. This study aimed to expand the utilisation of 3D Slicer further by using it to visualise, segment, and grade UTUCs. The 3D Slicer version 5.2.2 for macOS, built on 22/02/2023, was downloaded and installed for this study.

*Orange® data mining software*

Orange® data mining (University of Ljubljana, Slovenia) is a freely available online tool (https://orangedatamining.com/) hosted by GitHub (version 3.35.0) and released under a GNU General Public License was downloaded and installed.

*Tumour Segmentation in 3D Slicer*

The methodology employed in this study encompassed a series of procedures to effectively handle DICOM images, perform 3D segmentation utilising the “grow-from-seeds” tool within the 3D Slicer software (version 5.2.2), and convert the DICOM slices into the widely adopted 3D Neuroimaging Informatics Technology Initiative (NIfTI) format. The “grow-from-seeds” tool facilitated 3D segmentation of the Region of Interest (ROI) housing the targeted tumour through the software’s integrated functionality. Manual seed placement was employed to ascertain the initial seed points within the tumour ROI, enabling the segmentation algorithm to expand the delineation by incorporating the provided seeds. Fine-tuning of segmentation parameters was performed iteratively to optimise the segmentation outcomes. The segmentation task was performed by an experienced radiologist (Reader 1) and an expert urosurgical oncologist (Reader 2). Both experts refined the tumour boundaries of all slices. A dice-score test with a threshold of 0.8 was applied to assess segmentation consistency. The top and bottom slices in CT for the tumours were omitted to avoid a partial volume effect. The histopathological evaluation following biopsy or nephrectomy was considered the reference (gold) standard. Overall, the segmentation process in this study was conducted to ensure accurate and precise delineation of the tumour boundaries, and an experienced medical professional confirmed the results.

*Feature selection*

Eight feature selection methods, including information gain, gain ratio, Gini index, Least Absolute Shrinkage and Selection Operator (LASSO), reliefF, fast Correlation based filter, Analysis of Variance (ANOVA), and recursive feature elimination, were utilised to identify relevant features. The backward feature elimination using shape and cross-validation was employed to select the optimal feature set. The process involved estimating SHAP feature importance, removing low-importance features, and visualising the results through plots. This approach facilitated effective feature selection based on their impact on model performance. Python was utilised for feature selection. To oversample the low-grade class, the Synthetic Minority Oversampling Technique (SMOTE) platform was applied.

**Supplementary Material S2. Tumour Segmentation Workflow**

**
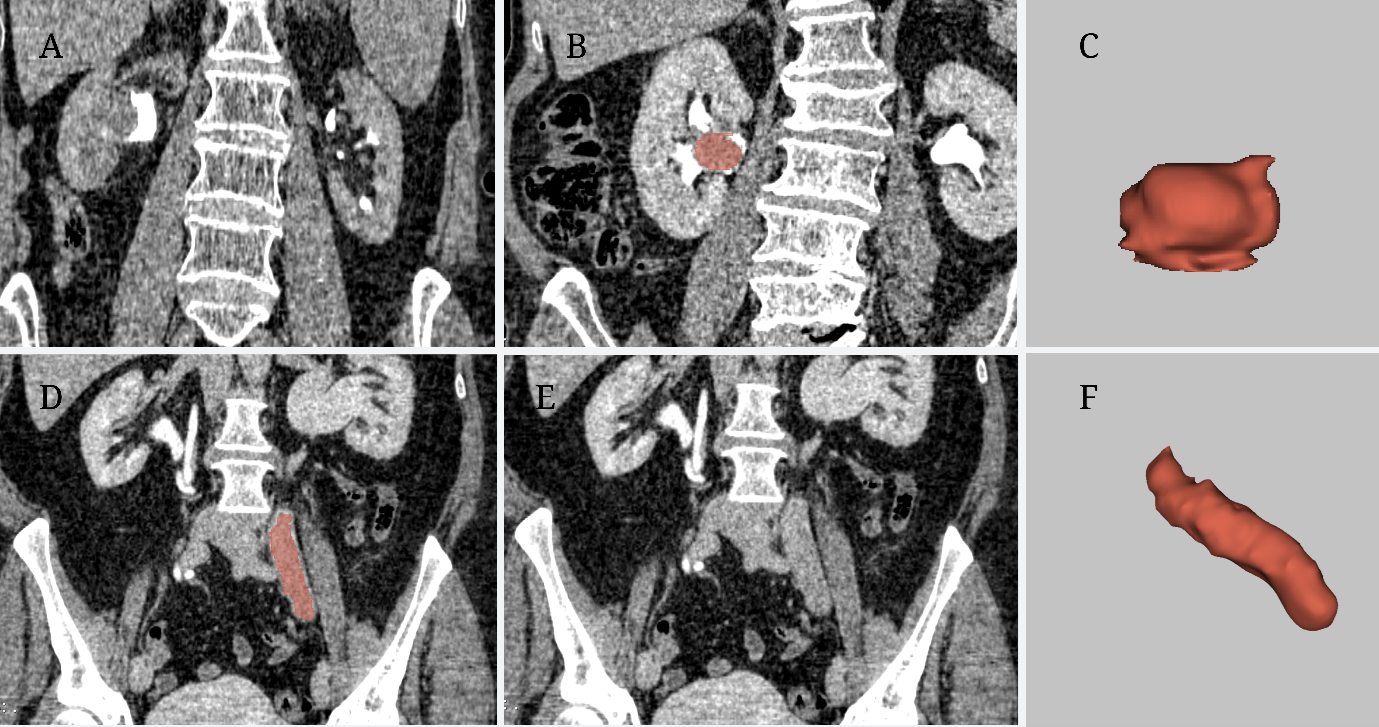
**

***Figure S2.*** *Graphics showing CT urogram and 3D segmentation in two UTUC patients done in 3D Slicer. For Patient 1, a male with a right-sided renal pelvis tumour (210 mm), panels (A) and (B) show the tumour without and with a segmentation mask. The 3D representation (C) displays a tumour volume of 6.92 cm³. For Patient 2, a female with a left-sided mid-ureter tumour (50 mm), images (D) and (E) provide views without and with the mask. The 3D model (F) demonstrated a volume of 11.39 cm³. The figure emphasises the integration of tumour size, volume, and location into the experimental workflow.*

**Supplementary Material S3. Results Overview**

*Radiomics models for discrimination of high and low grades*

Eleven radiomics models were employed to discriminate between high- and low-grade UTUC or early and advanced TNM stages. Fast correlation-based filter feature selection resulted in 11 features comprising clinical variables, radiomics features, and their combinations. The models were evaluated using ROC AUC, sensitivity, specificity, f1-score, precision, and recall as accuracy metrices. ANOVA analysis revealed that the combined grade dataset significantly outperformed the radiomics grade dataset (mean F-statistic=5.86, *p*=0.02) and the clinical grade dataset (mean F-statistic=11.46, *p*=0.003). Additionally, the combined grade dataset showed statistically significant (*p*<0.05) superiority in sensitivity (mean F-statistic=2.21, *p*=0.15), specificity (mean F-statistic=2.59, *p*=0.12), f1-score (mean F-statistic=2.33, *p*=0.14), and recall (mean F-statistic=3.38, *p*=0.08) compared to the other datasets. Notably, the radiomics grade dataset achieved the highest precision with statistical significance (mean F-statistic=12.4, *p*=0.002). The combined stage dataset was the best among all the evaluated metrices.

The box plot visually compared all UTUC grade datasets, highlighting the combined grade dataset as the best performer in the median, inter-quartile range, and outliers. It allowed for a quick and effective assessment of central tendency, variability, and data distribution across the different datasets (Figure 3A). Among the models, the random forest classifier performed the best for ROC AUC (*p*=0.002), sensitivity (*p*=0.002), specificity (*p*=0.0003), and f1-score (*p*=0.0007). However, when considering the p-values for the models, there was no statistically significant difference observed for ROC AUC (*p*=0.47), sensitivity (*p*=0.33), specificity (*p*=0.19), and f1-score (*p*=0.4). Consequently, our focus shifted towards utilising the combined data in this cohort for further analysis, aiming to effectively discriminate between high and low grades (Figure 3B). The MLP Classifier model achieved high precision (0.89), recall (0.89), and f1-score (0.89), with a significant difference in f1-score compared to other models (*p*=0.04). The model also had a high ROC AUC of 0.94, sensitivity of 84%, and specificity of 93%. Ureteroscopic biopsies predicted histological grade with an accuracy of 77.27%, sensitivity of 72.73%, and specificity of 86.36%. The match rates for low-grade and high-grade cases were 61.29% and 91.43%, respectively **Figure S3**.

*
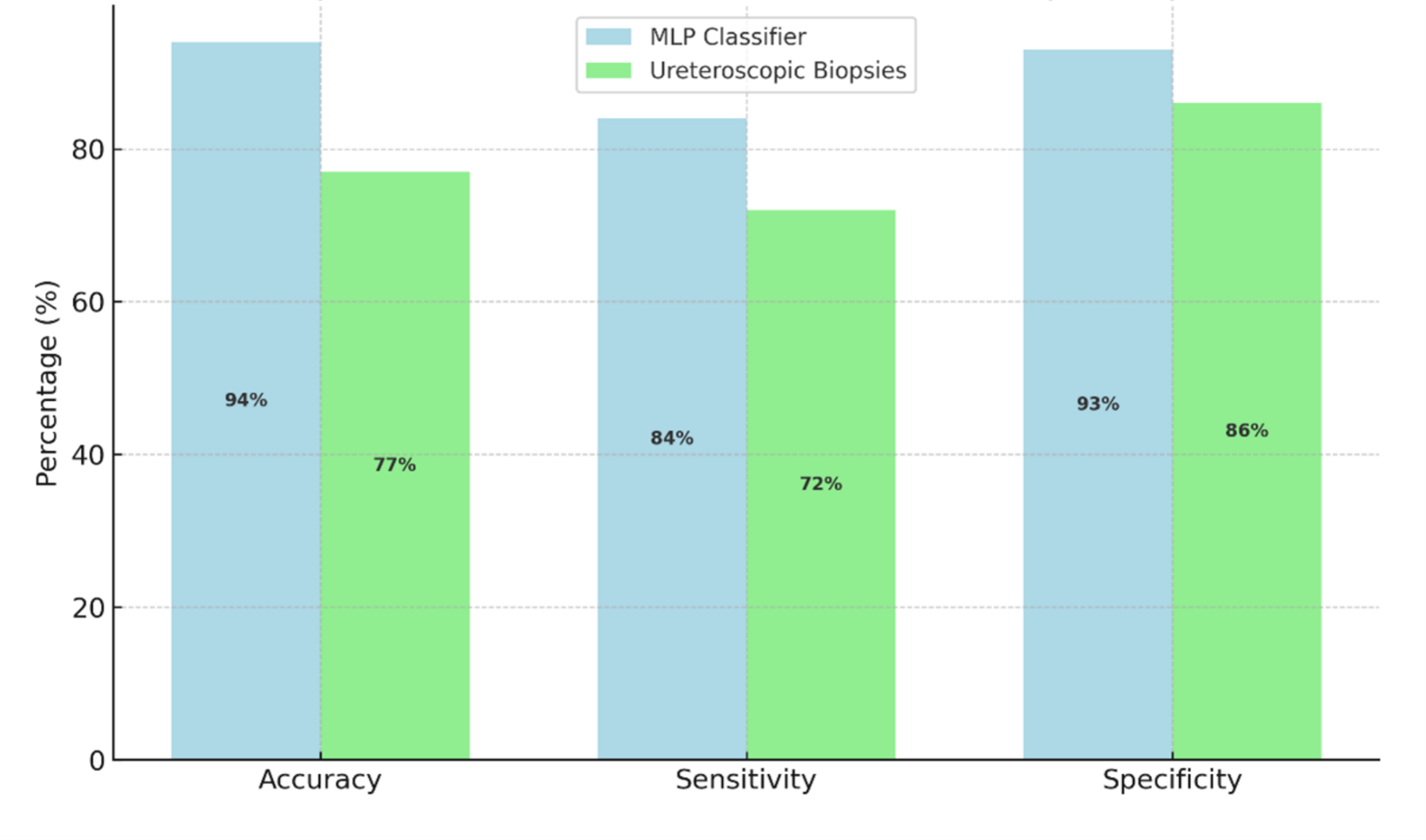
*

***Figure S3.*** *This bar chart provides a visual comparison between the performance metrics of the MLP Classifier model and Ureteroscopic Biopsies in the context of grading histological samples. The parameters compared include Accuracy, Sensitivity, and Specificity, crucial indicators of diagnostic efficacy.*

*Model evaluation*

Generating a confusion matrix showed the proportion of predicted values for the classes of interest (High/H and Low/L) compared to the actual values. The total number of actual values was 150, equalising 75 samples per class (H or L). Results revealed that the random forest classifier, on the other hand, demonstrated a sensitivity of 85.3% and a specificity of 86.6%, suggesting its strong performance in reliably detecting positive and negative cases (Table 2). Compared to other models, the decision tree classifier performed poorly, with a sensitivity of 76% and a specificity of 74%. The logistic regression model achieved 80% sensitivity and 89% specificity.

*Performance evaluation and features contribution analysis of the optimal grading prediction model*

The MLP Classifier model demonstrated high accuracy with a sensitivity of 92.86% and specificity of 80% on the testing set (Figure 4A). It achieved an AUC of 0.99 ± 0.00 on the training set and a strong AUC of 0.94 ± 0.06 on the testing set, indicating excellent discrimination and generalisation abilities (Figure 4B). The learning curve (Figure 4C) showed convergence and stabilisation of the training and test AUC scores, suggesting potential performance improvement with additional training data. The MLP Classifier achieved an AUC of 0.94 on the test subset (Figure 4D). The feature importance ranking based on SV (Shapley Value) highlighted wavelet-LLH_giszm_GrayLevelNonUniformityNormalized and stage as the most influential features for predicting UTUC grading. The Shapley Additive Explanations (SHAP) analysis provided insights into the significance of features. In the clinical category, the ‘Stage’ feature had a significant influence, while the body mass index had an inverse impact. Among the radiomics features, one attribute positively influenced the model’s prediction, while another had a negative contribution. The SHAP value impact plot summarised these relationships, enhancing our understanding of the model’s behaviour and potential for improvements.

*The superior performance of the combined stage dataset*

We evaluated 11 classification models on three datasets (clinical variables, radiomics, and combined). Recursive Feature Elimination with SHAPE and CV was used for feature selection (Figure 5A). The combined stage dataset showed superior discriminative ability in distinguishing between early and advanced stages compared to the clinical stage and radiomics stage datasets.

The mean scores of the three datasets were compared. The Clinical-stage had the highest mean score (M=0.72), followed closely by the Combined-stage (M=0.72) and then the radiomics stage (M=0.68). Statistical analysis revealed no significant difference between Combined-stage and Radiomics-stage (*p*=0.34), combined-stage and clinical-stage (*p*=1.0), but a marginal significance between Radiomics-stage and clinical-stage (*p*=0.1; Figure 5B). Among the models, the MLP Classifier exhibited strong performance with an average precision of 0.82, recall of 0.81, and F1-score of 0.81. It achieved an ROC AUC of 0.85, indicating good discriminative ability (Table 3). The classifier demonstrated high sensitivity (86.4%) and specificity (76.3%). Consequently, the research shifted towards analysing the combined data in this cohort to effectively differentiate between early and advanced stages (Figure 5C).

*Performance evaluation and features contribution analysis of the optimal staging prediction model*

The Logistic Regression model emerged as the most accurate and suitable for precision-oriented classification tasks. It achieved a sensitivity of 75.33% and specificity of 83.33% on the testing set, accurately identifying positive and negative cases (Figure 6A). The model demonstrated excellent discrimination and generalisation abilities with a perfect AUC of 0.91 ± 0.01 on the training set and a strong AUC of 0.88±0.06 on the testing set (Figure 6B). The learning curve (Figure 6C) indicated that the model could benefit from additional training data to improve its performance, as the training and test AUC scores did not converge and stabilise yet. The feature importance ranking based on SV (Figure 6D) revealed that grade, CIS, and original_shape_Maximum2DDiameterColumn were the top three influential features for UTUC staging prediction. The SHAP analysis provided insights into the significance of each feature. In the clinical category, grade and CIS had a significant impact, while cytology had the opposite effect. Among the radiomics features, one feature had a strong positive influence, while others contributed negatively. The SHAP value impact plot (Figure 6D) reflected these relationships, enhancing our understanding and suggesting ways to improve the model’s performance.

*Hierarchical clustering*

The dendrogram displayed shows the results of applying hierarchical clustering to the high and low grades of UTUCs. The selected instances from the plot are highlighted in red, and the original data is displayed with an additional column indicating whether an instance is selected. The Hierarchical Clustering algorithm utilised the Average linkage method to measure distances between clusters, which computes the average distance between the elements of two clusters, as shown in **Figure S4**.

**
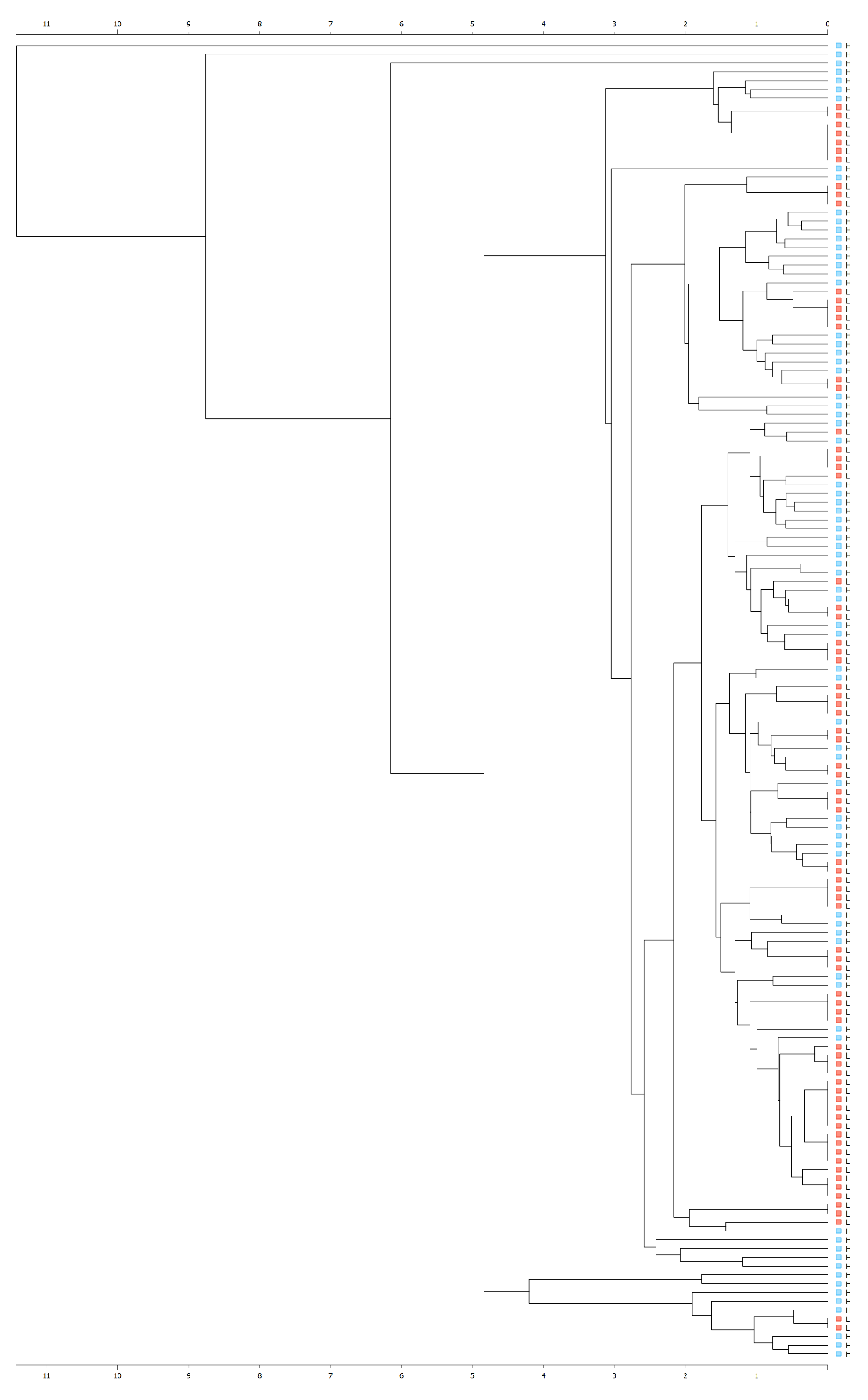
**

***Figure S4.*** *The dendrogram shows hierarchical clustering on the pathological grades of UTUCs.*
